# Doppler echocardiographic indices in aortic coarctation: a comparison of profiles before and after stenting

**DOI:** 10.5830/CVJA-2012-044

**Published:** 2012-10

**Authors:** Shokoufeh Hajsadeghi, Seyed-Mohammad Fereshtehnejad, Mahshid Ojaghi, Hossein Ali Bassiri, Mohammad Reza Keramati, Mitra Chitsazan, Saeid Gholami

**Affiliations:** Department of Cardiology, Rasoul-e-Akram Hospital, Tehran; University of Medical Sciences (TUMS), Tehran, Iran; Firoozgar Clinical Research Development Center (FCRDC), Firoozgar Hospital, Tehran University of Medical Sciences (TUMS), Tehran, Iran; Department of Echocardiography and Cardiology, Shaheed Rajaie Cardiovascular Medical and Research Centre, Tehran; University of Medical Sciences (TUMS), Tehran, Iran; Department of Echocardiography and Cardiology, Shaheed Rajaie Cardiovascular Medical and Research Centre, Tehran; University of Medical Sciences (TUMS), Tehran, Iran; Department of Surgery, Firoozgar Hospital, Tehran; University of Medical Sciences (TUMS), Tehran, Iran; Medical Students’ Cardiology Research Centre, Shaheed; Rajaie Cardiovascular Medical and Research Centre, Tehran; University of Medical Sciences (TUMS), Tehran, Iran; Medical Students’ Research Committee (MSRC), Tehran; University of Medical Sciences (TUMS), and Rasoul-e-Akram Hospital, Tehran, Iran

**Keywords:** aortic coarctation, Doppler echocardiography, index, diagnostic values

## Abstract

**Background:**

Diagnosis of aortic coarctation is important as it is a difficult condition to evaluate, especially in adults. A Doppler echocardiographic index could provide a simple tool to evaluate coarctation. This study was performed to compare Doppler echocardiographic profiles before and after stenting and to assess the diagnostic value of a complete list of echocardiographic indices for detecting aortic coarctation.

**Methods:**

This prospective study was conducted on 23 patients with a diagnosis of aortic coarctation based on angiography. Echocardiographic assessment was done twice for all patients before and after stenting. Each time, two-dimensional and Doppler echocardiographic imaging modalities were performed and complete lists of indices were recorded for each case. After comparing the values of indices before and after stenting, diagnostic values of each index were calculated in order to diagnose significant coarctation.

**Results:**

Twenty-three patients, including 16 males and seven females with a mean age of 26.14 ± 10.17 years, were enrolled in this study. Except for the mean velocity and mean pressure gradient of the abdominal aorta, the values of the other indices of the abdominal/descending aorta showed enough change after stenting to indicate significant diagnostic accuracy for detecting aortic coarctation. The velocity–time integral and the pressure half-time were among the indices with the highest accuracy rates for this purpose (all *p* < 0.001).

**Conclusion:**

Post-stenting echocardiographic profiles could provide a reliable reference value of the normal aortic haemodynamics as a unique identification of each patient and it is presumed that these indices could be used as reliable indicators of response to treatment.

## Abstract

As one of the most common congenital heart defects,^1^ aortic coarctation has a wide morphological spectrum that varies from transverse arch and isthmal hypoplasia, which are seen most commonly in new-born babies, to discrete stenosis or membrane-like obstructions, which are typically observed in older patients.^2^ Aortic coarctation presenting during adult life most frequently represents cases either of re-coarctation following previous transcatheter or surgical therapy, or missed cases of native coarctation.^3^

Nowadays, percutaneous stenting is an accepted form of treatment for isolated coarctation of the aorta.^4^ Balloon-expandable endovascular stents have been used in various locations since the 1980s.^5^-^15^ Stents support the integrity of the vessel wall after balloon dilation by opposing the recoil of the elastic vascular stenosis and re-adhering the torn intima to the media. This minimises the extension of wall tears and subsequent dissection or aneurysm formation.^7^,^11^,^13^,^15^,^16^ Moreover, stents provide a homogenous framework for smooth endothelial growth along the aortic wall that reduces the risk of thrombosis, neo-intimal hyperplasia and subsequent restenosis.^5^

Diagnosis and evaluation of coarctation is of great importance, not only before stenting but also after implantation, in order to assess the occurrence of restenosis. With regard to evaluation of aortic coarctation, cardiovascular magnetic resonance (CMR) imaging is the procedure of choice.^17^ However, its use may be limited because of lack of availability or clinical contraindications.

On one hand, due to the wide availability, in the presence of clinical suspicion of aortic coarctation, echocardiography is the only available bedside diagnostic tool. It is also used in the initial assessment and follow up after the intervention of patients with coarctation. On the other hand, two-dimensional and colour Doppler echocardiographic techniques including analysis of pulse-wave and continuous-wave Doppler across the coarctation site and at the abdominal aorta are also used for the indirect evaluation of coarctation.^4^ Therefore a Doppler echocardiographic index, independent of cardiac function or other lesions and based on two-dimensional measurements of the transverse aortic arch would provide a simple tool to improve the accuracy of diagnosing coarctation.

Previous investigations have produced a few echocardiographic indices, which were validated in a limited group of patients, to evaluate the condition of patients with aortic coarctation.^4^,^18^-^20^ However, the age groups of the patients differed and the various echocardiographic indices assessed were incomplete. Therefore we aimed to evaluate the changes in a complete list of echocardiographic profiles in patients with aortic coarctation before and after stenting, and to determine the diagnostic value of these indices as an indicator of aortic coarctation.

## Methods

This prospective study was conducted on 23 consecutive patients with the diagnosis of aortic coarctation who were referred to Rajaei Heart Centre (affiliated to Tehran University of Medical Sciences), Tehran, Iran, from April 2008 to August 2009. For this purpose, 40 patients with a definite diagnosis of aortic coarctation, based on an angiographic study, were referred to the ECHO Research Centre of the hospital for further assessment.

All cases had primary unoperated coarctation. Patients with other concomitant lesions, including aortic stenosis or regurgitation, patent ductus arteriosus, anomalies of the head and neck vessels, and long-segment aortic coarctation or hypoplastic arch were excluded.

According to their lesions, these 40 patients were candidates for balloon angioplasty, and stenting of the aortic coarctation was performed on all of them. Among these patients, 23 who had been proven to have no gradient and residual stenosis at the time of stenting were enrolled into the study.

In addition to baseline and demographic variables (e.g. age, gender, blood pressure and length of stenosis), the characteristics of stenting, including length and width of stent, length and width of balloon, and before- and after-stent peak gradient of the catheter were recorded for all patients. All gradients were directly measured during catheterisation.

Informed written consent was obtained from all patients. The research project was approved by the ethics committee of Tehran University of Medical Sciences.

## Echocardiographic evaluation

Echocardiographic assessment was done twice on all patients, 24 hours before and 24 hours after stenting. Two-dimensional and Doppler echocardiographic imaging studies were performed using a Vivid 3 Imaging System (GE, USA) in accordance with institutional guidelines. All echocardiographic studies were done by one echocardiologist before and after stenting.

Both the abdominal and descending aorta were evaluated during Doppler echocardiography. The standard suprasternal position was used to measure the maximum velocity across the coarctation site and then continuous-wave Doppler recordings were obtained. Pulsed-wave Doppler from the standard subcostal view was also performed to document the flow pattern of the abdominal aorta.

**Fig. 1.**
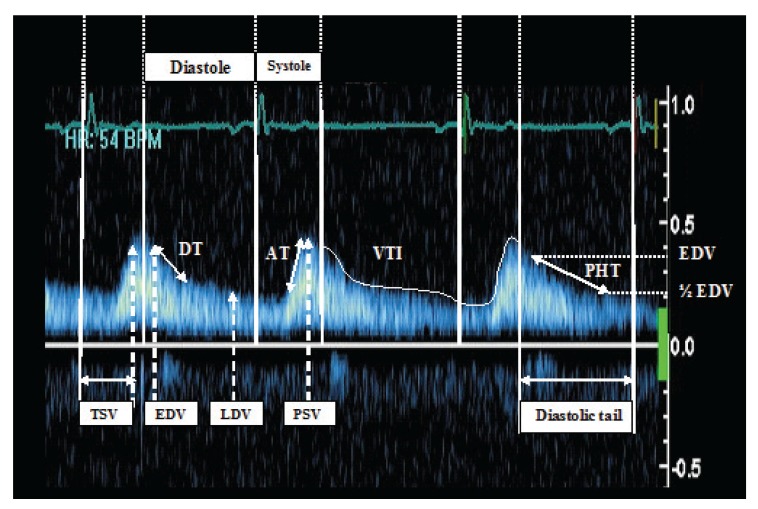
Continuous-wave Doppler echocardiography of the abdominal aorta. AT : systolic acceleration time is measured from the onset of the systolic upstroke to the systolic peak. DT: deceleration time is measured from Peak E velocity to the point where the slope of the slowing flow would intercept the baseline. EDV: early diastolic velocity: maximum diastolic velocity on early diastole. LDV: late diastolic velocity: maximum diastolic velocity on late diastole. PHT: pressure half-time (of diastole) is the time interval for the peak diastolic pressure gradient to be reduced by one half. PSV: peak systolic velocity: maximum systolic velocity. TSV: time to peak systolic velocity: time beginning from onset of QRS complex to peak systolic velocity. VTI: velocity time integral: the area under the curve, shown in both systole and diastole.

**Fig. 2.**
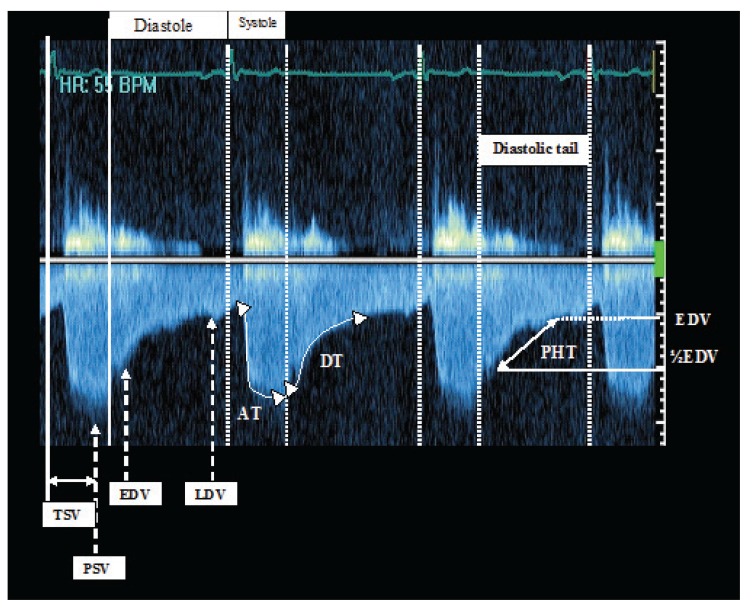
Continous-wave Doppler echocardiography of descending aorta. AT : systolic acceleration time, DT: deceleration time, EDV: early diastolic velocity, LDV: late diastolic velocity, PHT: pressure half-time (of diastole), PSV: peak systolic velocity, TSV: time to peak systolic velocity.

The measurements obtained in the abdominal and/or descending aorta were peak systolic velocity (PSV) (m/s), early diastolic velocity (EDV) (m/s), late diastolic velocity (LDV) (m/s), systolic acceleration time (AT) (m/s), pressure half-time (PHT) (m/s), mean velocity (m/s), mean of peak gradient (mean PG), diastolic velocity/systolic velocity (D/S ratio velocity), velocity–time integral (VTI), time to peak systolic velocity (m/s), pulse delay, and pulsatility index (PI). Samples of pulse-wave Doppler echocardiography of the abdominal and descending aorta are shown in Figs 1 and 2, respectively.

All studies, including pre- and post-stenting profiles, were performed with simultaneous electrocardiographic monitoring (ECG based), and the onset of diastole was assumed at the end of the electrocardiographic T wave. Moreover, three measurements were taken from three consecutive cycles and the averaged values were calculated and reported as the main records.

Intra- and inter-observer variability of the measurements were checked and calculated on a sample of patients by means of re-measurement of systolic and diastolic velocities by the same observer (intra-observer variability) and a second blinded observer (inter-observer variability). The calculated mean percentage error or disagreement was so low as to be considered negligible. Pre-stenting values of the Doppler echocardiographic profile were assumed to represent so-called ‘significant coarctation’, while post-stenting data corresponded with ‘no coarctation’.

## Definitions

Echocardiographic indices performed were defined as follows:

• Deceleration time (DT) was measured from peak E velocity to the point where the slope of the slowing flow would intercept the baseline.^21^
• Systolic acceleration time was measured from the onset of the systolic upstroke to the systolic peak.^21^
• Pressure half-time (PHT) was the time interval for the peak pressure gradient to be reduced by one half (PHT = 0.29 × DT).^21^
• Abdominal aortic pulse delay was quantified by measuring the time to peak velocity in the abdominal aorta and comparing it with the same value measured from flow at the aortic annulus. This value should be indexed to the heart rate by dividing the absolute value by the square root of the PR interval.^21^
• Pulsatility index was the systolic velocity minus diastolic velocity divided by the mean velocity $\left[\frac{\;systolic\;velocity\;–\;diastolic\;velocity}{mean\;velocity}\right]$.^22^
• Early diastolic velocity (EDV) was maximum diastolic velocity in early diastole.
• Late diastolic velocity (LDV) was maximum diastolic velocity in late diastole (atrial contraction).^23^
• Peak systolic velocity was maximum systolic velocity.^24^
• Velocity time integral was the area under the velocity curve.
• Time to peak systolic velocity was the time from onset of the QRS complex to peak systolic velocity measured by pulsewave Doppler echocardiography.
• Pulse delay index was calculated by means of the following equation: $\frac{Time\;to\;peak\;systolic\;velocity\;of\;LVOT\;–\;Time\;to\;peak\;systolic\;velocity\;of\;abdominal\;aorta}{\surd RRinterval}$

## Statistical analysis

Data were analysed using SPSS v 17 software (Chicago, IL, USA). For each of the measured variables or indices, descriptive values were expressed as the mean ± SD for normally distributed variables, and median and interquartile range (IQR) in the case of non-normal distributions. All data were initially analysed using the Kolmogorov–Smirnov test to assess for normality. The within-group changes of each Doppler echocardiographic index were evaluated using the paired *t*-test. Pearson’s correlation analysis was also used to assess the relationship between baseline aortic gradient measured by catheter and Doppler echocardiographic indices.

Receiver operating characteristic curve (ROC) analysis was performed to assess the predictability of significant coarctation (pre-stenting condition) with the quantitative indices of the study, and then to compare area under the curve (AUC) of these variables. For this purpose, the first measured profiles before stenting were considered to be the values of patients with significant aortic coarctation, while the next measured indices after stenting were taken as the profiles of the individuals without coarctation.

The cut-off points were then determined in each ROC analysis. The best predictive cut-off value was the one that gave the highest sensitivity and specificity simultaneously. The diagnostic values of each cut-off point, including sensitivity and specificity, were calculated and reported. All *p*-values were two-tailed and *p* < 0.05 was considered statistically significant.

## Results

Twenty-three patients, including 16 (69.6%) males and seven (30.4%) females with a mean age of 26.14 ± 10.17 years (range 14–56) were enrolled in this study. The median time since diagnosis of disease was 12.00 (IQR = 31) months and the mean length of the stenosis was 20.44 ± 10.47 mm. In addition, the mean baseline ejection fraction was 54.55 ± 5.10% (range 35–60%) and 16 ± 69.6% of patients were hypertensive. All baseline and stenting characteristics of the patients are listed in Table 1.

**Table 1.** Baseline And Stenting Characteristics Of The Patients

Patients’ age (years)	26.14 ± 10.17
Patients’ gender (%)	
Male	16 (69.6)
Female	7 (30.4)
Blood pressure	
Systolic blood pressure (mmHg)	158.18 ± 24.18
Diastolic blood pressure (mmHg)	85.23 ± 10.96
Time since diagnosis (months)	12.00 (IQR = 31)
Aortic valve (%)	
BAV	13 (56.5)
TAV	10 (43.5)
Ejection fraction (%)	54.55 ± 5.10
Length of the balloon (mm)	18.55 ± 4.44
Width of the balloon (mm)	5.350 ± 3.95
Length of the stent (mm)	37.16 ± 3.67
Width of the stent (mm)	8.47 ± 1.94
Peak before-stenting gradient of the catheter (mmHg)	57.05 ± 12.69
Peak after-stenting gradient of the catheter (mm Hg)	2.38 ± 5.39
Before-stenting gradient of the catheter (%)	85.00 ± 7.69
After-stenting gradient of the catheter (%)	14.17 ± 9.96
Length of the stenosis (mm)	20.44 ± 10.47

All values for continuous variables are mean ± SD except for time since diagnosis, which is median (IQR).

The patients underwent Doppler echocardiography at the time of enrolment and after stenting. All Doppler echocardiographic profiles of the abdominal and descending aorta (before and after stenting) are given in Table 2. The differences between the two measurements were calculated and mean percentages are listed in Table 2.

**Table 2.** Doppler Echocardiographic Profile Of Abdominal And Descending Aorta Before And After Stenting (In Percentage Change Column, Positive Values Show Reduction Whereas Negatives Show Increase In Values After Stenting)

*Doppler echocardiographic profile*	*Mean values before stenting*		*Mean values after stenting*		*Mean percentage change after stenting (%)*			
	*Abdominal aorta*	*Descending aorta*	*Abdominal aorta*	*Descending aorta*	*Abdominal aorta*	p*-value*	*Descending aorta*	p*-value*
PSV (m/s)	0.58 ± 0.13	3.84 ± 0.77	0.77 ± 0.20	2.41 ± 0.51	–37.60 ± 35.37	< 0.001	34.88 ± 18.83	< 0.001
EDV (m/s)	0.42 ± 0.15	1.93 ± 0.67	0.21 ± 0.10	0.56 ± 0.33	44.76 ± 32.92	< 0.001	65.04 ± 29.45	< 0.001
LDV (m/s)	0.27 ± 0.09	0.87 ± 0.51	0.19 ± 0.07	0.29 ± 0.14	22.83 ± 32.91	0.002	61.45 ± 27.27	< 0.001
AT (m/s)	229.07 ± 75.48	101.30 ± 56.11	102.14 ± 34.47	68.99 ± 26.32	50.35 ± 23.33	< 0.001	12.45 ± 64.18	0.020
PHT (m/s)	196.39 ± 91.86	163.68 ± 82.72	61.00 ± 19.54	57.26 ± 27.24	59.51 ± 29.14	< 0.001	48.50 ± 62.49	< 0.001
Mean velocity (m/s)	0.38 ± 0.10	1.86 ± 0.45	0.35 ± 0.10	0.96 ± 0.24	1.00 ± 35.74	< 0.001	45.15 ± 21.28	< 0.001
Mean PG	0.69 ± 0.33	19.81 ± 7.96	0.70 ± 0.44	6.58 ± 3.08	–26.95 ± 86.39	0.932	57.81 ± 36.62	< 0.001
D/S ratio velocity	0.72 ± 0.24	0.50 ± 0.15	0.27 ± 0.13	0.22 ± 0.10	59.68 ± 19.65	< 0.001	48.54 ± 37.37	< 0.001
VTI	31.96 ± 9.41	152.85 ± 56.39	24.02 ± 6.90	58.65 ± 17.59	18.34 ± 33.39	0.005	57.37 ± 19.97	< 0.001
Time to peak systolic velocity (m/s)	370.61 ± 76.40	239.59 ± 43.47	243.35 ± 36.05	203.30 ± 41.56	31.48 ± 17.40	< 0.001	11.00 ± 27.40	0.026
Pulse delay	–	8.11 ± 3.36	–	3.19 ± 1.78	–	–	54.62 ± 26.91	< 0.001
Pulsatility index	–	0.89 ± 0.30	–	1.75 ± 0.51	–	–	–119.57 ± 110.16	< 0.001

PSV: peak systolic velocity, EDV: early diastolic velocity, LDV: late diastolic velocity, AT: systolic acceleration time, PHT: pressure half-time, PG: peak gradient, D/S: diastolic velocity/systolic velocity, VTI: velocity time integral. All *p*-values are from paired *t*-test and *p* < 0.05 is considered significant.

Stenting decreased the pulse-delay index from 8.11 ± 3.36 to 3.19 ± 1.78 (*p* < 0.001). Additionally, significant reductions were noted in the EDV (*p*_Ab_ < 0.001, *p*_Ds_ < 0.001), LDV (*p*_Ab_ = 0.002, *p*_Ds_ < 0.001), AT (*p*_Ab_ < 0.001, *p*_Ds_ = 0.020), PHT (*p*_Ab_ < 0.001, *p*_Ds_ < 0.001), mean velocity (*p*_Ab_ < 0.001, *p*_Ds_ < 0.001), mean PG (*p*_Ds_ < 0.001), D/S ratio velocity (*p*_Ab_ < 0.001, *p*_Ds_ < 0.001), VTI (*p*_Ab_ = 0.005, *p*_Ds_ < 0.001) and time to peak systolic velocity (*p*_Ab_ < 0.001, *p*_Ds_ = 0.026). While the PSV of the abdominal aorta was significantly increased after stenting (*p*_Ab_ < 0.001), the corresponding value of the descending aorta had decreased (*p*_Ds_ < 0.001). The largest percentage increase (119.57%) was achieved in the pulsatility index, which increased from 0.89 ± 0.30 to 1.75 ± 0.51 (*p*_Ds_ < 0.001).

The ROC curve analysis was performed to evaluate the diagnostic values of different Doppler echocardiographic indices in order to differentiate significant coarctation (pre-stenting condition) from post-stenting conditions or without coarctation. Diagnostic values of all 12 Doppler echocardiographic indices in both the abdominal and descending aorta are given in Table 3.

**Table 3.** Diagnostic Values Of Different Cut-Off Points Of Echocardiographic Profiles Of Abdominal And Descending Aorta To Differentiate Significant Coarctation (Pre-Stenting Condition) From Post-Stenting Condition Or Without Coarctation (All Data Derived From ROC Curve Analysis)

*Echocardiographic index*	*AUC*	p*-value*	*Cut-off point*	*Sensitivity (%)*	*Specificity (%)*
Abdominal aorta					
PSV (m/s)	0.816	< 0.001	0.655	73.9	73.9
EDV (m/s)	0.902	< 0.001	0.325	82.6	87
LDV (m/s)	0.747	0.004	0.205	69.6	65.2
AT (m/s)	0.940	< 0.001	131.00	91.3	87
			146.50	82.6	91.3
PHT (m/s)	0.960	< 0.001	83.5	91.3	82.6
			106.00	87	100
Mean velocity (m/s)	0.616	0.177	-	-	-
Mean PG	0.560	0.489	-	-	-
D/S ratio velocity	0.968	< 0.001	0.43	95.7	87
VTI	0.757	0.003	27.5	73.9	69.6
Time to peak systolic velocity (m/s)	0.953	< 0.001	308.5	82.6	100
Descending aorta					
PSV (m/s)	0.938	< 0.001	3.09	90.9	87
			3.37	81.8	100
EDV (m/s)	0.965	< 0.001	0.96	90.9	87
LDV (m/s)	0.922	< 0.001	0.385	90.9	82.6
AT (m/s)	0.696	0.025	77.43	68.2	69.6
PHT (m/s)	0.900	< 0.001	85.5	81.8	87
Mean velocity (m/s)	0.969	< 0.001	1.18	95.5	87
			1.33	90.9	91.3
Mean PG	0.949	< 0.001	10.50	90.9	91.3
D/S ratio velocity	0.931	< 0.001	0.365	81.8	91.3
VTI	0.980	< 0.001	87.50	90.9	91.3
			94.5	86.4	100
Time to peak systolic velocity (m/s)	0.698	0.023	242.50	50	91.3
Pulse delay	0.957	< 0.001	5.98	87	95.7
Pulsatility index	0.945	< 0.001	1.21	87	91.3

PSV: peak systolic velocity, EDV: early diastolic velocity, LDV: late diastolic velocity, AT: systolic acceleration time, PHT: pressure half-time, PG: peak gradient, D/S: diastolic velocity/systolic velocity, VTI: velocity time integral, AUC: area under curve.

As shown in Table 3, except for the mean velocity (*p*_Ab_ = 0.177) and mean PG (*p*_Ab_ = 0.489) of the abdominal aorta, other indices of the abdominal or descending aorta had a statistically significant area under the curve (AUC) to distinguish patients with significant aortic coarctation or a pre-stenting condition. The VTI of the descending aorta had the greatest AUC of 0.980 (*p*_Ds_ < 0.001) such that the velocity–time integral of > 87.50 had 90.9% sensitivity and 91.3% specificity, and the values of > 94.50 had 86.4% sensitivity and 100% specificity for detection of significant aortic coarctation.

**Fig. 3.**
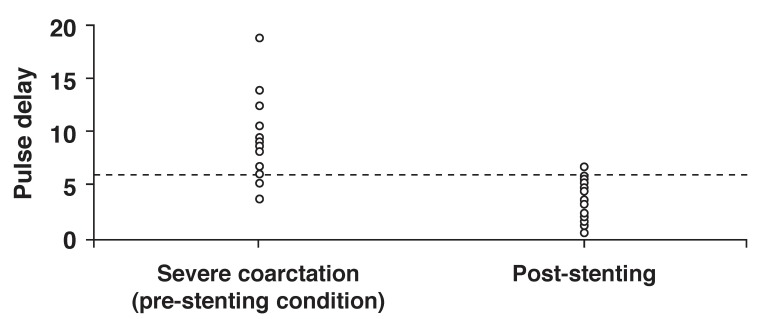
Cut-off points of 5.98 for pulse delay of descending aorta to differentiate significant coarctation (prestenting condition) from post-stenting condition or without coarctation.

**Fig. 4.**
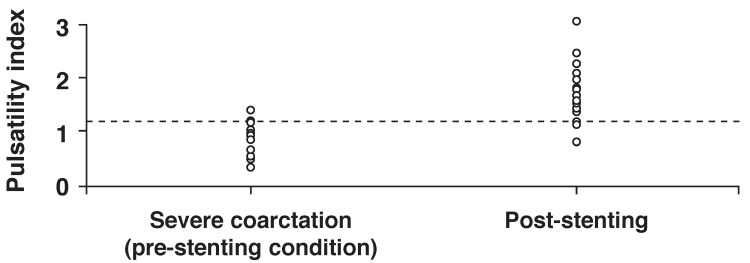
Cut-off points of 1.21 for pulsatility index of descending aorta to differentiate significant coarctation (pre-stenting condition) from post-stenting condition or without coarctation.

A pulse delay of > 5.98 had a sensitivity of 87% and specificity of 95.7% to diagnose significant aortic coarctation (Fig. 3). Moreover, as illustrated in Fig. 4, a pulsatility index of > 1.21 had 87% sensitivity and 91.3% specificity to differentiate significant coarctation (pre-stenting condition) from the post-stenting condition or without coarctation.

As shown in Table 4, the baseline peak aortic gradient measured by catheter was significantly correlated with some of the pre-stenting echocardiographic profiles of the abdominal and descending aorta. In both the abdominal and descending aorta, the strongest correlation of the peak gradient was observed with pressure half-time as a direct relationship (*r*_Ab_ = 0.637, *p*_Ab_ = 0.001; *r*_Ds_ = 0.613, *p*_Ds_ = 0.003). The velocity–time integral of the descending aorta was also directly correlated with before-stenting peak gradients (*r*_Ds_ = 0.548, *p*_Ds_ = 0.010). Other correlations are shown in Table 4.

**Table 4.** Evaluation Of The Correlation Between Severity Of Coarctation (Baseline Peak Gradient Of The Catheter) And Pre-Stenting Echocardiographic Profiles Of Abdominal And Descending Aorta

*Echocardiographic index*	*Correlation coefficient (r)*	*p-value*
Abdominal aorta		
PSV (m/s)	–0.093	0.681
EDV (m/s)	0.087	0.701
LDV (m/s)	–0.026	0.909
AT (m/s)	0.014	0.952
P.H.T (m/s)	0.637	0.001
Mean velocity (m/s)	0.066	0.769
Mean PG	0.115	0.610
D/S ratio velocity	0.054	0.811
VTI	0.446	0.038
Time to peak systolic velocity (m/s)	–0.004	0.987
Descending aorta		
PSV (m/s)	0.265	0.245
EDV (m/s)	0.474	0.030
LDV (m/s)	0.592	0.005
AT (m/s)	0.093	0.688
PHT (m/s)	0.613	0.003
Mean velocity (m/s)	0.473	0.030
Mean PG	0.436	0.048
D/S ratio velocity	0.374	0.095
VTI	0.548	0.010
Time to peak systolic velocity (m/s)	–0.020	0.931
Pulse delay	–0.075	0.739
Pulsatility index	0.084	0.710

PSV: peak systolic velocity, EDV: early diastolic velocity, LDV: late diastolic velocity, AT: systolic acceleration time, PHT: pressure half-time, PG: peak gradient, D/S: diastolic velocity/systolic velocity, VTI: velocity time integral, AUC: area under curve. All data derived from two-sided Pearson correlation analysis.

The possible correlation of the baseline peak aortic gradient with the mean percentage change in echocardiographic profiles after stenting was also evaluated. Our findings showed a reverse correlation between severity of coarctation and changes in LDV after stenting. The higher the gradient, the lower the change in the LDV of the abdominal aorta (*r*_Ab_ = –0.455, *p*_Ab_ = 0.033). By contrast, changes in the PHT of the abdominal aorta were directly correlated with the baseline catheter gradient (*r*_Ab_ = 0.436, *p*_Ab_ = 0.043). Changes in the other echocardiographic indices were not significantly correlated with the baseline aortic gradient.

## Discussion

Coarctation of the aorta is characterised by anatomical obstruction in the descending aorta. It is difficult to evaluate this obstruction because of the variability in cardiac output, number and size of collaterals, and peripheral resistance.^25^ Primary clinical diagnosis and subsequent assessment of the severity of coarctation and re-coarctation of the aorta have traditionally been made based on the judgment of the character of the femoral pulse. Also known as a secondary event, absent, weakened or delayed femoral pulses occur as a result of obstruction in aortic coarctation.

The pressure drop across the obstruction (the gradient), pressure half-time, and diastolic flow are widely used but inaccurate indices to diagnose aortic coarctation. They can be affected by many other factors such as cardiac output,^26^ lesion length,^27^ the presence of collateral networks,^26^ and aortic compliance.^28^

Stent implantation has been used as a reliable treatment for coarctation of the aorta. It has several advantages, rendering it superior to angioplasty alone.^29^ The effect of stents on blood flow dynamics are not well known. Moreover, despite the importance of close follow up to evaluate complications and the long-term effect on the blood pressure of these patients, there are no adequate long-term follow-up indices for these patients.

Therefore the present study was carried out to find reliable, quantitative Doppler echocardiographic indices for assessment of the severity of coarctation of the aorta before stenting and comparing these indices with the post-stenting condition. This would provide a valuable profile to indicate successful stent implantation.

All previous methods, including monitoring the blood pressure, two-dimensional echocardiography, cardiac magnetic resonance (CMR) and angiography have failed to give favourable results. Persistent hypertension, even in the absence of a recurrent or residual stenosis,^25^,^30^ insufficient anatomical evaluation of two-dimensional echocardiography,^31^ and disrupted MRI by metallic artifacts (or noise) have limited the value of these indices to assess the patient at post-intervention follow up.^32^ Furthermore, angiography as an invasive procedure has known complications.

Doppler echocardiography overcomes these problems in the follow up of such patients. However, echocardiography may be less sensitive than angiography, spiral computed tomography and MRI in detecting aneurysms after stent placement.^32^

Based on our results, the Doppler echocardiographic profile was found to be valid for differentiating significant coarctation from the normal condition (after stenting), with high diagnostic values. As demonstrated in the results, continuous flow was significantly decreased from before to after stenting in both the descending and abdominal aorta. Moreover, monophasic systolic flow was shown to increase significantly after stenting. In comparison with a few similar studies,^4^,^20^ we assessed more indices, which we will discuss below.

According to our results, aortic pulse delay decreased after stenting. The results also showed that a pulsatility index of < 1.21 was suggestive of significant coarctation of the aorta. This cut-off point was calculated as < 2 in a study by Silvilairat *et al*.^33^ Currently, it is known that obstructed blood flow due to aortic coarctation leads to pressure drop and loss of the pulse wave distal to the stenosis. This can be observed by echocardiography typically as decreased pulsatility of the abdominal aorta after cardiac systole.^34^

Early and late diastolic velocities were found to be significant markers in the assessment of the severity of coarctation. In addition, mean peak gradient of the descending aorta was significantly reduced by as much as 58% following stenting. This could be the result of changed flow dynamics along the stent.^4^ However, in some patients, there was an under- or overestimation of the pressure gradient across the coarctation site on Doppler echocardiography. As mentioned, these are affected by other factors, such as cardiac output,^26^ lesion length,^27^ the presence of collateral networks,^26^ and aortic compliance.^28^ Therefore pressure gradient alone as an index of aortic narrowing is often inadequate.

Although the mean velocity in both the descending and abdominal aorta significantly decreased after stenting, the difference was more significant in the descending aorta, with an approximately 45% reduction. Similarly, the acceleration time in the descending aorta was different from the corresponding measurement in the ascending aorta in coarctation.^31^ This is manifested clinically by radial femoral delay and diminished pulses distal to the coarctation. After stent implantation, the acceleration time showed statistically significant decreases in both the descending and abdominal aorta.

Based on our findings, the velocity–time integral and time to peak systolic velocity can be also used as new markers of significant coarctation. Both indices significantly decreased after stenting. We also found pressure half-time indices (systolic and diastolic velocity half-times, systolic and diastolic pressure half-times) can be used to assess the severity of coarctation, with sensitivities of 87 and 81.8% and specificities of 100 and 87% for the abdominal aorta and descending aorta, respectively. These findings were in keeping with the results of previous investigations by Carvalho *et al.*^35^ and Tan *et al.*,^4^ which reported a significant effect of coarctation of the aorta on these indices. A study by Lim and Ralston however was in disagreement with regard to systolic indices.^36^

Diastolic velocities (DVs) and diastolic pressure decays have been shown to provide invaluable information for assessing the severity of coarctation.^35^-^37^ The index of D/S ratio velocity was first used by Tan *et al*.^4^ as a marker of significant coarctation. They demonstrated that a D/S ratio velocity of > 0.53 had a sensitivity of 100% and specificity of 96% for detecting significant aortic coarctation. They believed that by correlating diastolic with systolic velocity, this ratio would be less affected by variations in heart rate, stroke volume, systemic blood pressure and aortic compliance.^4^

However, we found a lower cut-off point for D/S ratio velocity. The D/S ratio of > 0.365 in the descending aorta had a sensitivity of 95.7% and specificity of 87%, whereas the ratio of > 0.43 in the abdominal aorta had a sensitivity of 81.8% and specificity of 91.3% in defining significant coarctation of the aorta. D/S ratio velocity appears to be a good marker of this condition. Recently, the D/S ratio as a non-invasive measurement of coronary flow velocity has been used to evaluate left anterior descending artery (LAD) stenosis. By contrast with the aorta, the D/S ratio was found to be significantly lower in patients with more critical stenosis of the LAD.^38^

Besides evaluation of the diagnostic value of the echocardiographic indices, a correlation analysis was also performed in our study to assess the relationship between the severity of coarctation before stenting and the echocardiographic indices. As shown, PHT and VTI of the abdominal aorta and EDV, EDV, PHT, mean velocity and mean peak gradient of the descending aorta correlated significantly with the peak gradient in the coarctation site, measured by catheterisation prior to stent implantation.

In addition, the higher pre-stenting gradients were associated with lower changes in LDV of the abdominal aorta, while changes in PHT of the abdominal aorta were directly correlated with the baseline gradient. The observed correlation between the baseline severity of coarctation and the changes in PHT after stenting leads us to conclude that this index (PHT) is probably the best to determine stenting outcome and the probable occurrence of restenosis. Nevertheless, it should be evaluated in further studies.

Although our study had some limitations, including small sample size, no long-term follow up and no CMR imaging for evaluation of the aortic coarctation index, it had some findings which have not been reported before. One must also consider the problem of using a functional technique to predict anatomical obstruction, especially in adults where the haemodynamics are directly and significantly affected by the presence and extent of collateral blood flow. Doppler techniques are more valuable in neonates with coarctation, where collateral flow has not had time to develop, and the haemodynamic consequences are more clearly related to the anatomical obstruction.

The results of the present study showed that a complete set of Doppler echocardiographic profiles could potentially provide a valid method to diagnose significant aortic coarctation. Velocity–time integral, time to peak systolic velocity, systolic acceleration time and mean velocity were sensitive and specific enough to detect significant aortic coarctation, as were peak systolic, early diastolic and late diastolic velocities, pressure half-time, peak gradient and D/S ratio velocity, which were validated in previous studies.

To the best of our knowledge this is the first evaluation of such a complete list of Doppler echocardiographic indices to detect significant coarctation of the aorta. Our findings emphasise the advantages of Doppler echocardiography for close monitoring of patients with aortic coarctation. Although these echocardiographic indices do improve dramatically in patients who undergo stenting, they never return to normal values even if no residual stenosis exists.

## Conclusion

We found a significant difference between pre- and post-stenting echocardiographic values, which could provide valuable insight for evaluation of follow up and response to treatment in patients with aortic coarctation. Post-stenting echocardiographic profiles of each patient could therefore provide an individualised and reliable reference value of his/her normal aortic haemodynamics, and early detection of restenosis could be achieved by comparison of post-stenting values with follow-up values. A similar clinical approach is used in echocardiographic follow up of patients with prosthetic heart valves.^39^

We suggest that these echocardiographic indices could be used as reliable detectors of the patient’s response to treatment, and their predictive role in the follow up of these patients is obvious. However, in order to evaluate recurrent stenosis, it would be important to re-evaluate patients during longer-term follow up, which could be undertaken in further studies.
